# The Impact of Cross-Cultural Adaptation on the Psychology and Entrepreneurial Intention of Venture Entrepreneurs

**DOI:** 10.3389/fpsyg.2021.705075

**Published:** 2021-10-07

**Authors:** Long Ye, Xu-Yang Dong

**Affiliations:** ^1^School of Foreign Studies, Shaoguan University, Shaoguan, China; ^2^College of Foreign Studies, Jiaxing University, Jiaxing, China

**Keywords:** cultural psychology, cross-cultural adaptation, venture entrepreneur, entrepreneurial intention, psychological pressure

## Abstract

The purpose was to integrate cultural psychology into cross-cultural adaptation and analyze the factors of entrepreneurial psychology and entrepreneurial intention of venture entrepreneurs. The research framework of cross-cultural adaptation is constructed, and four hypotheses are put forward, and 100 venture entrepreneurs in multinational enterprises are randomly recruited and investigated through the QS (Questionnaire Survey) design. Finally, the results are analyzed through statistical software. The results show that among the basic information of venture entrepreneurs, the proportion of gender is balanced, and the educational level is generally high, with the majority of young entrepreneurs aged 20–35. Meanwhile, there are significant differences in the four dimensions of cross-cultural competence, cross-cultural adaptation, entrepreneurial intention, and psychological adaptation of venture entrepreneurs (*p*<0.05). Length of service influences cross-cultural competence, cross-cultural adaptation, and entrepreneurial intention but does not affect psychological adaptation. Hence, cross-cultural adaptation has a great impact on the entrepreneurial intention and psychological adaptation of venture entrepreneurs and provides a practical basis for the entrepreneurial optimization of venture entrepreneurs.

## Introduction

Driven by market competition and supported by state policies, China’s entrepreneurship and innovation activities are booming, and innovative enterprises have become a major force to stimulate employment, innovation, and economic growth (Shim et al., 2016). The year 2015 witnessed the appearance of a new term with distinctive characteristics of times, namely, innovation and entrepreneurship, which has been frequently mentioned in government policies and documents ever since. Meanwhile, as the mainstay of China’s technological innovation, a substantial number of high-quality technology-based SMEs (small- and medium-sized enterprises) have emerged under the wave of mass innovation and entrepreneurship (Salter and Mckelvey, 2016; Zhang et al., 2016). According to the dynamic data released by the torch center of the Ministry of science and technology, in the first quarter of 2020 alone, over 9,500 technology-based SMEs have got the national accreditation and have successfully entered the warehouse, and the number is still increasing (Eng et al., 2020).

By entering the overseas market, Chinese enterprises provide new opportunities and challenges for cross-cultural adaptation research (Chung et al., 2018). As the main entrepreneurial force, venture entrepreneurs play a unique role in the promotion of entrepreneurial performance, which has always been a hot topic in the entrepreneurship field. Scholars have studied venture entrepreneurs from the perspectives of human capital, social capital, and personality psychology (Braun et al., 2017). Particularly, entrepreneur’s psychology is considered to be the core drive of entrepreneurial activities and achieve success, which provides ideas to entrepreneurial talents cultivation for successful entrepreneurship. With the increasing number of foreign-funded enterprises and Sino-foreign joint ventures, venture entrepreneurs are frequently faced with adaptation challenges from cross-cultural work and life environments. Moreover, under different enterprise histories and experiences, diverse social cultures, complex geopolitics, and language barriers, venture entrepreneurs feel strong psychological pressure (Cestari et al., 2016). New ventures also encounter various problems during transnational operation, for example, how they understand their cross-cultural partners well and how they adjust their behavior to enhance their entrepreneurial intention (Martin et al., 2016; Russo et al., 2020).

Islam et al. (2020) believed that entrepreneurial intention determined the result of enterprise activities, and the result was closely related to the strategic layout of the enterprise and the work adaptability of employees. Neneh (2020) suggested that entrepreneurs’ self-efficacy and cultural adaptation could alleviate psychological pressure and enhance entrepreneurial performance. Some scholars have also explored the relationship between various cross-cultural adaptation combinations and venture enterprises’ invisible performance and intention, which significantly influences the development of new enterprises and the competitiveness of enterprises (Rogoza et al., 2018). Under this background, it is crucial to analyze the relationship between the entrepreneur’s cross-cultural adaptation, psychological adaptation, and entrepreneurial intention.

In short, based on cultural psychology and theoretical analysis, a research framework of cross-cultural adaptation is proposed for venture entrepreneurs, and research hypotheses are put forward and are verified through QS (Questionnaire Survey) to provide strategies for venture entrepreneurs to adapt to the cross-cultural environment and improve work efficiency. The innovation is to analyze the adjustment mechanism of cross-cultural adaptation, entrepreneur psychology, entrepreneurial intention, and entrepreneurial performance, which is of great significance for venture entrepreneurs to formulate cross-cultural strategies and manage enterprises.

## Related Work

Foreign studies on transnational entrepreneurs focus on cross-cultural psychology and international human resources management, and the research on cross-cultural psychology focuses on the cross-cultural adaptation factors of transnational entrepreneurs (Feng and Chen, 2020; Zaman et al., 2021). In cross-cultural adaptation research, the definition of adaptation includes two dimensions: psychological adaptation and socio-cultural adaptation, which is widely used in the field of cross-cultural psychology. Helling and Chandler (2021) revealed that psychological adaptation was emotional satisfaction and well-being. Socio-cultural adaptation referred to the acquisition of skills to integrate into and successfully respond to the new culture, which was reflected in behavioral changes. Additionally, psychological adaptation was based on stress-coping theory, measured *via* individual stress, depression, life satisfaction (Fendt-Newlin et al., 2020).

Since the beginning of the 21st century, in foreign countries, cross-cultural adaptation was still investigated mainly through quantitative research based on the empirical test, though innovations have been made in research perspectives, and more moderating variables are introduced (Christofi et al., 2021). For example, Lee et al. (2020) proposed that under the moderating effects of corporate support and cultural distance, cross-cultural adaptation had an impact on entrepreneurial intention. Some scholars also explored the relationship between individual factors, such as gender, educational background, self-efficacy, and cross-cultural adaptation, through literature review (Wen et al., 2020). By contrast, domestic researchers paid more attention to the impact of cross-cultural adaptation. Specifically, Tubadji et al. (2021) showed that the relationship between cultural intelligence, cultural novelty, and cross-cultural adaptation was the inverted U-shaped curve. Meanwhile, cross-cultural management practice and cultural adaptation had a significantly positive correlation, in which organizational support played a mediating role, while cross-cultural adaptation had a great impact on entrepreneurs’ entrepreneurial intention and entrepreneurial performance.

In summary, the research on the cross-cultural adaptation factors of transnational entrepreneurs should be based on the theory of cultural psychology, thereby analyzing the correlation between cross-cultural adaptation, psychological adaptation, and entrepreneurial intention of transnational entrepreneurs.

## Methodology

### Theories of Cultural Psychology

Cultural psychology is one of the latest research fields of psychology, which studies the relationship between psychology and culture and reveals the mechanism of the integration of culture and psychology (Lee, 2020). Meanwhile, cultural psychology is an extension of the scientific view of mainstream psychology. Mainstream psychology tries to establish an empirical and objective research system based on a natural science model, in which the research objects and research method are separated from the cultural basis, while human factors are ignored for a scientific facade. Thus, the pursuit of objectivity replaces the pursuit of truth (Bedford and Yeh, 2020; Eronen and Romeijn, 2020). The test chart of cultural psychology is free from the shackles of naturalism science and restores the cultural character of psychology. Here, it is believed that, based on the richness and complexity of human psychological phenomena (Hong and Francis, 2020), psychological research on human psychology and behavior cannot be separated from specific social culture and personal cultural background. The combination of social culture and personal culture is embodied in the people’s social practices, which proves that cultural psychology puts human psychology into the specific social, cultural, historical, and practical framework for understanding (Fisher, 2020). In terms of research methods, besides qualitative research (Pratt et al., 2020), the authenticity of the research object is ensured, thus making the research results more consistent with the actual situation and showing a high ecological validity (Deng et al., 2021; Kourtesis et al., 2021). Additionally, cultural diversity and psychological diversification are the consensus of psychology (Dichter et al., 2016; Moreno et al., 2020), which is a requirement of cultural psychology to promote equal culture exchange. Here, the complex psychological phenomenon of venture entrepreneurs is mainly studied, and their commonness and differences are pointed out.

### Theories of Cross-Cultural Adaptation

**Figure 1:**
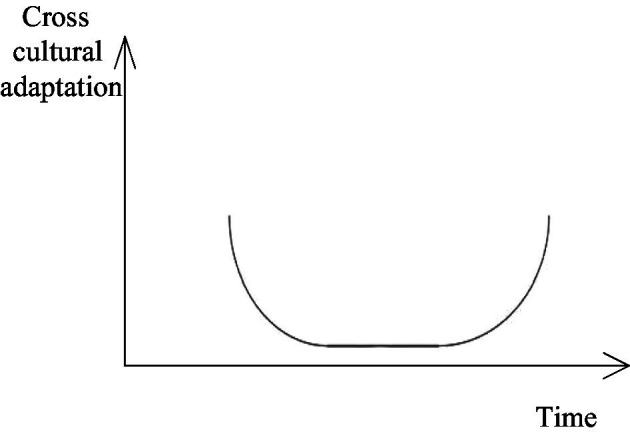
U-shaped emotional curve.

Firstly, cultural shock and the U-shaped curve hypothesis should be introduced (Murshed et al., 2021). Cultural shock refers to the anxiety over the sudden loss of familiar social communication symbols (Xia, 2020), which is caused by the loss of familiar situations and meanings in the original social life and communication. The cross-cultural adaptation usually goes through three phases: initial adjustment, crisis and re-adaptation, and the U-shaped emotional curve of emotional adaptation, as shown in Figure 1.

**Figure 2:**
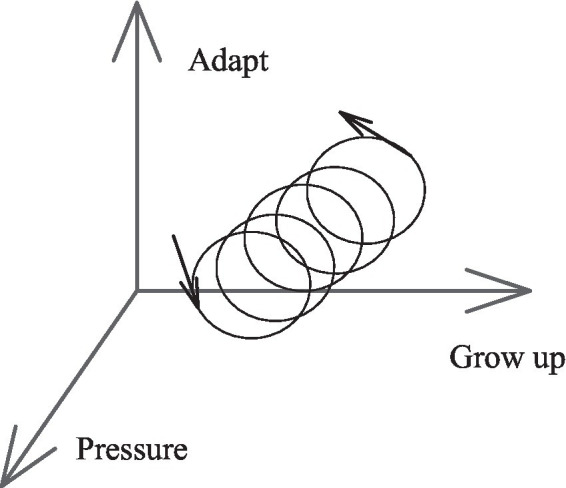
Pressure-adaptation-dynamic-growth model.

Secondly, the integration theory of cross-cultural adaptation is discussed (Gün, 2020). The theory involves three core concepts: cross-cultural adaptation, communication, and stranger. Cross-cultural adaptation is a dynamic process. A person establishes and maintains a relatively stable, mutually beneficial, and functional relationship with the new environment, reinterprets a new culture, and constructs a pressure-adaptation-dynamic-growth model, as shown in Figure 2.

Figure 2 describes the nature of the cross-cultural adaptation process consistent with the experience of strangers. The adaptation process causes psychological pressure, which is not a disease but a force to drive strangers to overcome difficulties, take the initiative to learn and adapt to the new culture, and ultimately, help achieve individual growth. This process presents a spiral dynamic trajectory.

**Figure 3:**
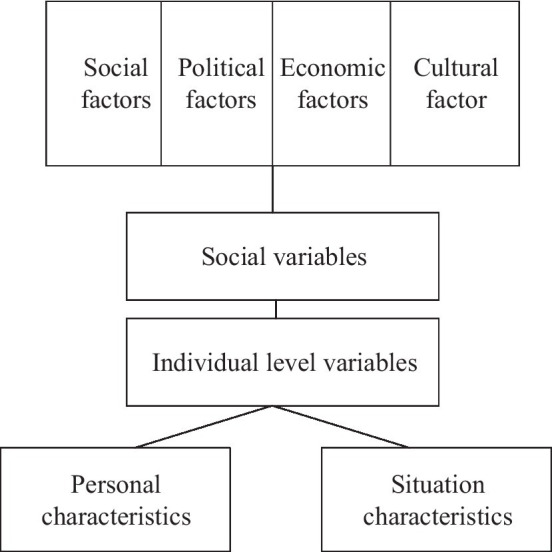
The process of cross-cultural adaptation.

Finally, the theory of the cross-cultural adaptation process is presented (Dåderman et al., 2020). After the U-curve hypotheses are constructed, a process model for cross-cultural adaptation is proposed, as shown in Figure 3.

Individuals will cope with psychological pressure from emotion, behavior, and cognition and learn new social and cultural skills, thereby achieving psychological and socio-cultural adaptation. Many factors at the individual and social levels will affect the pressure response and social skill acquisition process of cross-cultural adaptation. The macro and micro factors of cross-cultural adaptation are considered in the proposed model, along with psychological adaptation and social-cultural adaptation, providing a comprehensive reference framework for understanding the process of cross-cultural adaptation. Accordingly, the mechanism and results of cross-cultural adaptation are analyzed for some Chinese multinational entrepreneurs.

**Figure 4:**
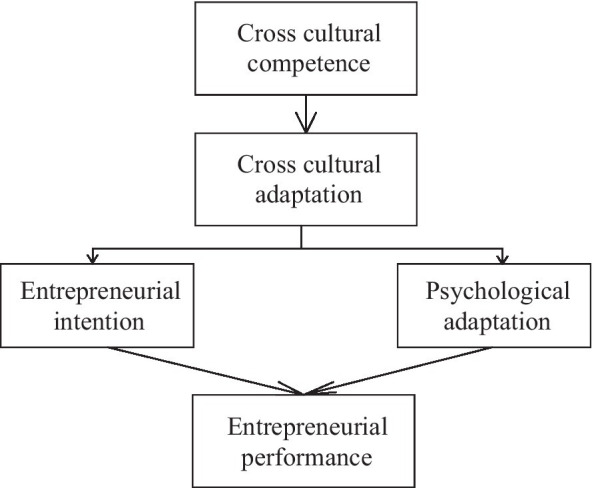
Research framework of cross-cultural adaptation.

Based on the above theories, the cross-cultural competence of venture entrepreneurs is taken as the independent variable, cross-cultural adaptation is taken as the mediating variable, entrepreneurial performance and psychological adaptation are taken as the dependent variables, and entrepreneurial intention is taken as the moderating variable of cross-cultural adaptation and psychological adaptation. Thus, the research framework is proposed for cross-cultural adaptation, as shown in Figure 4.

Based on the proposed model, four theoretical hypotheses are put forward.

*H1*: Cross-cultural competence is positively correlated with entrepreneurial performance.

*H2*: Cross-cultural adaptation is positively correlated with psychological adaptation.

*H3*: Cross-cultural adaptation is positively correlated with the entrepreneurial intention of venture entrepreneurs.

*H4*: Entrepreneurial intention positively moderates the relationship between cross-cultural adaptation and entrepreneurial performance.

### QS and Data Analysis

1. Here, the items of the QS are scored using the Likert scale (Omotayo et al., 2020). Then, the QS is divided into five scales, namely, cross-cultural adaptation, entrepreneurial intention, and entrepreneurial adaptation, and personal basic information, as shown in Tables 1–4, respectively

**Table 1:** Cross-cultural competence measurement.

Dimensions	Main contents
A_1_	Can you tolerate strong uncertainty caused by different cultural backgrounds?
A_2_	Are you willing to communicate with people from different cultural backgrounds?
A_3_	Under different cultural backgrounds, can you compromise on what you do not like?
A_4_	Do you often reflect upon the conversation with people from different cultural backgrounds?

**Table 2:** Cross-cultural adaptation measurement.

Dimensions	Main contents
B_1_	Can you quickly adapt to the role of cross-cultural entrepreneurs?
B_2_	Can you quickly adapt to the performance requirement of cross-cultural enterprises?
B_3_	Can you quickly adapt to the cultural atmosphere of cross-cultural enterprises?

**Table 3:** Entrepreneurial intention measurement.

Dimensions	Main contents
C_1_	Do you want to establish your own business?
C_2_	Do you have detailed entrepreneurial planning and ideas?
C_3_	Do you understand the relevant knowledge and detailed process of entrepreneurship?

**Table 4:** Psychological adaptation measurement.

Dimensions	Main contents
D_1_	Do you think you possess the psychological fitness to accomplish tasks efficiently?
D_2_	Do you think you have a high self-identity when you finish your tasks?
D_3_	Do you think work gives you a sense of belonging?

2. Here, 20 new and transnational ventures in China are selected through web page search. Meanwhile, 100 venture entrepreneurs who occupy managing positions and above are randomly chosen from the 20 new ventures. Afterward, 100 electronic QSs are issued, and 91 are recovered, with a recovery rate of 91%. SPSS26.0 statistical software is used for data analysis, and the validity and consistency of the scale are tested. The Pearson correlation analysis is chosen to test the correlation among the variables of cross-cultural competence, cross-cultural adaptation, entrepreneurial intention, psychological adaptation, and venture performance. Subsequently, following the single-factor ANOVA, the influence of gender, educational background, length of service, and enterprise nature on the above variables are analyzed. The reliability and validity of the QS are tested by SPSS26.0. KMO test is employed to check the correlation and partial correlation between variables, finding the value to be between 0 and 1. In practice, the KMO statistics should be above 0.7 to get a satisfactory result. If KMO statistics is less than 0.5, factor analysis should not be used, and the variable structure should be redesigned or other statistical analysis methods should be considered. In Bartlett’s spherical test, the independent factor analysis of each variable is invalid if the correlation matrix is a unit matrix. After the SPSS test, if Sig. < 0.05 (namely, *p*<0.05), the standard is met, the data are spherically distributed, and each variable is inter-independent. Cronbach’s α coefficient is most commonly used in reliability tests. Specifically, Cronbach’s α coefficient of the total scale is best to be above 0.8, and 0.7–0.8 is acceptable. Cronbach’s α coefficient of subscale is best to be above 0.7, and 0.6–0.7 is acceptable. If Cronbach’s α coefficient is less than 0.6, the QS should be redesigned.

## Results and Discussion

### Descriptive Analysis of Research Objects

**Figure 5:**
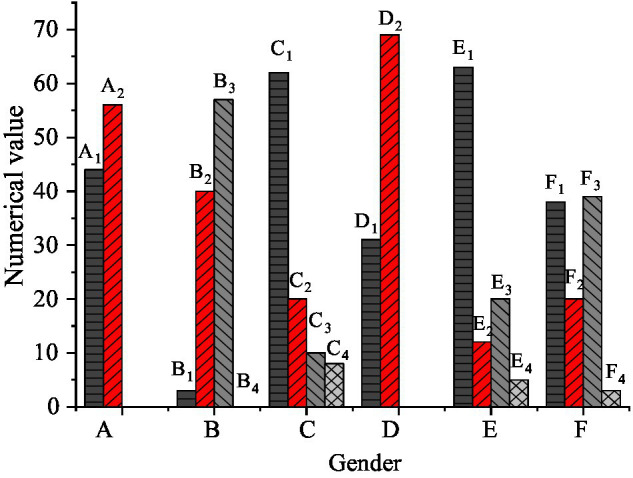
Descriptive statistical results of subjects’ basic information (a: Gender, a1: Male, a2: Female, b: Education, b1: Junior college and below, b2: Bachelor, b3: Master, b4: Doctor, c: Length of service, c1: Less than one-year, c2: 1–5 years, c3: 5–10 years, c4: 10–20 years, d: Marital status, d1: Married, d2: Unmarried, e: Age, e1: 20–35 years, e2: 35–45 years, e3: 45–55 years, e4: over 55 years, f: Corporate nature, f1: Sino-foreign joint venture, f2: Foreign enterprises, f3: Chinese enterprises).

The basic information of 100 subjects is described and counted, and the results are shown in Figure 5.

Figure 5 illustrates that the number of male and female venture entrepreneurs varies little, with 44 males and 56 females. Three of them have a junior college degree or below, 40 of them have a bachelor’s degree, 56 of them have a master’s degree, and five of them have a doctor’s degree, indicating that these venture entrepreneurs have a higher educational level. There are 31 married and 69 unmarried venture entrepreneurs. Meanwhile, 63 of the totals are aged between 20 and years old, indicating that these venture entrepreneurs are young. The number of Sino-foreign joint ventures and Chinese enterprises is 38 and 39, respectively, with almost the same proportion, indicating that the nature of venture enterprises is diverse.

The statistical results of Figure 5 may attribute to that with the improvement of China’s national education and economy, the educational level of young people also increases. Moudrý and Thaichon (2020) point out that under rapid social development, the employment rate of female youth has ascended, and their intention to start a business has strengthened significantly, which accounts for the even gender distribution of venture entrepreneurs. Most unmarried young people will start their businesses supported by the state’s entrepreneurial policies, and women are also given more opportunities to realize self-values.

### Reliability and Validity of the QS

**Figure 6:**
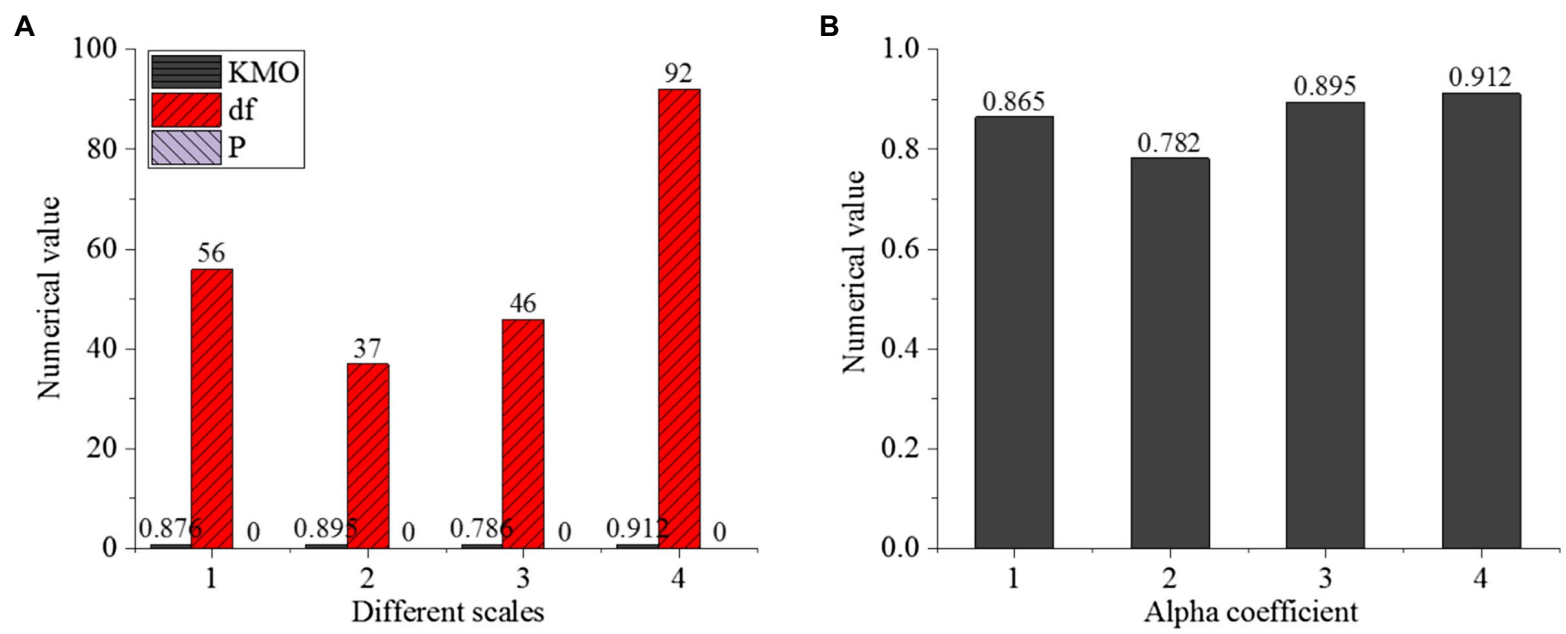
Validity test of research scales (**A**: different scales with Numerical value, **B**: Alpha coefficient with Numerical value, A: Cross-cultural competency scale, B: Cross-cultural adaptation scale, C: Entrepreneurial intention scale, D: Psychological adaptation scale of venture entrepreneurs).

The reliability and validity of the research scale are tested, as shown in Figure 6.

Figure 6 shows that the KMO (Kaiser-Meyer-Olkin) value of the cross-cultural competency scale is 0.876>0.8 and *p*<0.05. The KMO value of the cross-cultural adaptation scale is 0.895>0.8 and p<0.05. The KMO value of the entrepreneurial intention scale is 0.786>0.7, between 0.7 and 0.8, and p<0.05. The KMO value of the psychological adaptation scale of venture entrepreneurs is 0.912>0.8, and p<0.05. It shows that the validity of Bartlett’s spherical test is good and can be analyzed and tested in-depth. Among the four scales, the α coefficient of the three scales is higher than 0.8, indicating that the reliability is good. The reliability of the fourth scale is between 0.7 and 0.8, indicating that the designed problems of each scale can accurately describe the relevant contents.

### Correlation Analysis of Each Scale Variable

The correlation among the four variables of cross-cultural competence, cross-cultural adaptation, entrepreneurial intention, and psychological adaptation is shown in Table 5.

**Table 5:** The correlation between the variables.

	Cross-cultural competency	Cross-cultural adaptation	Entrepreneurial intention	Psychological adaptation
Cross-cultural competency	1			
Cross-cultural adaptation	0.473^**^	1		
Entrepreneurial intention	0.462^**^	0.586^**^	1	
Psychological adaptation	0.614^**^	0.601^**^	0.623^**^	1

** *p<0.01*.

Table 5 displays that from the perspective of cultural psychology, cross-cultural competence and cross-cultural adaptation have a significant relationship with a correlation coefficient of 0.473. The correlation coefficient between cross-cultural competence and entrepreneurial intention is 0.462. The correlation coefficient between cross-cultural competence and psychological adaptation of venture entrepreneurs is 0.614. The correlation coefficient between cross-cultural adaptation and entrepreneurial intention is 0.586. The coefficient of the relationship between cross-cultural adaptation and psychological adaptation of venture entrepreneurs is 0.601, which is significant. The correlation coefficient between entrepreneurial intention and psychological adaptation of venture entrepreneurs has a significant relationship, and the correlation coefficient is 0.623.

Table 5 indicates that for venture entrepreneurs, cross-cultural competence and cross-cultural adaptation are highly correlated with entrepreneurial intention and psychological adaptation. Besides, Goel and Karri (2020) proposed that the stronger the cultural adaptation of multinational entrepreneurs is, the less their psychological pressure is during working, which also proves that entrepreneurs’ psychological adaptation and cultural adaptation are strongly correlated. Only with good psychological adaptability and cross-cultural competence can they maintain their entrepreneurial intention in the process of cross-cultural adaptation and thus effectively improve entrepreneurial performance in a good psychological state.

### Analysis of Factors of Different Variables

**Figure 7:**
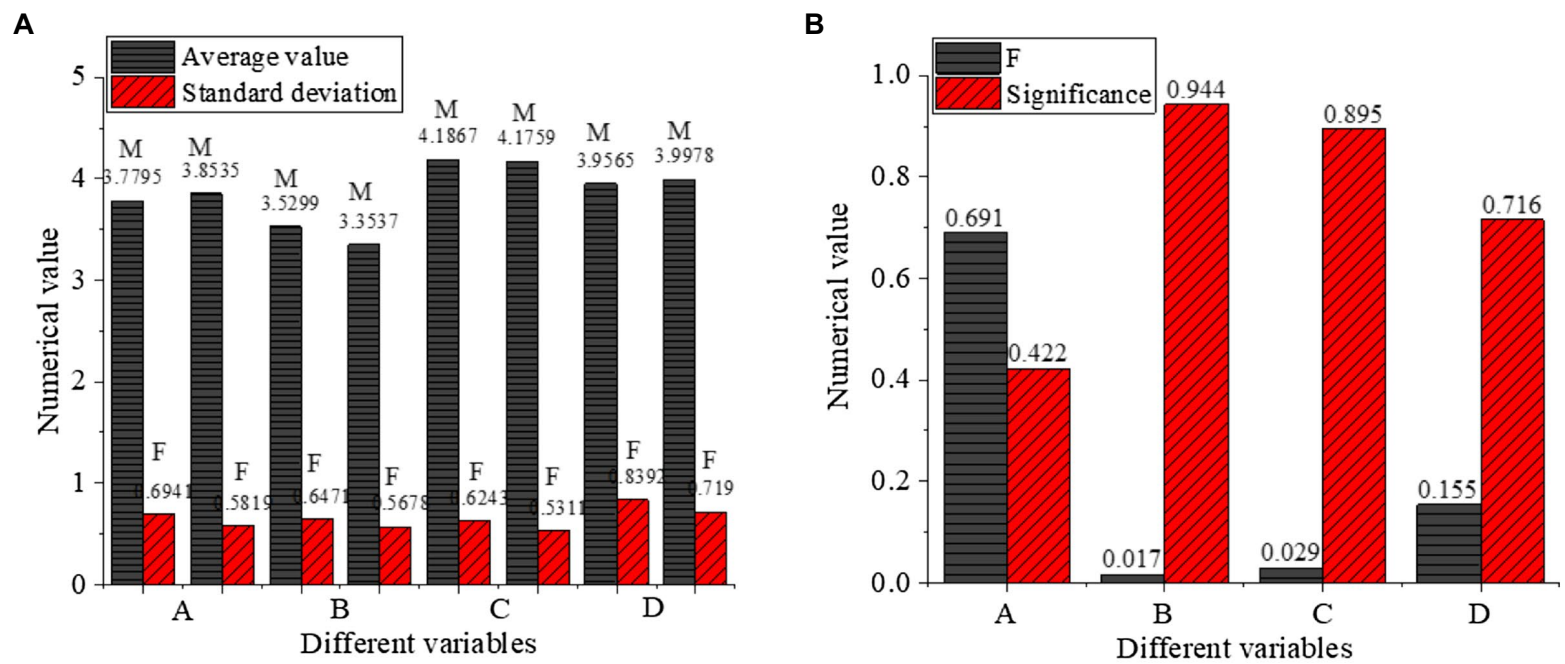
One-way ANOVA of gender (**A**: different variables with Numerical value, **B**: different variables with Numerical value, M: Male, F: Female, A: Cross-cultural competence, B: Cross-cultural adaptation, C: Entrepreneurial intention, D: Psychological adaptation of venture entrepreneurs).

Here, the influence is analyzed for gender, educational background, length of service, and enterprise nature on four variables: cross-cultural competence, cross-cultural adaptation, entrepreneurial intention, and psychological adaptation. The influence of gender on different variables is shown in Figure 7.

Figure 7 indicates that the average values of cross-cultural competence, cross-cultural adaptation, entrepreneurial intention, and psychological adaptation of male venture entrepreneurs are between [0.3527,4.1867] and that of the female is between [0.5317,0.8392]. The significance of cross-cultural competence, cross-cultural adaptation, entrepreneurial intention, and psychological adaptation of venture entrepreneurs in gender is 0.422, 0.944, 0.895, and 0.716, respectively, indicating that there is no significant difference in the four variables (cross-cultural competence, cross-cultural adaptation, entrepreneurial intention, and psychological adaptation) among venture entrepreneurs with different genders.

**Figure 8:**
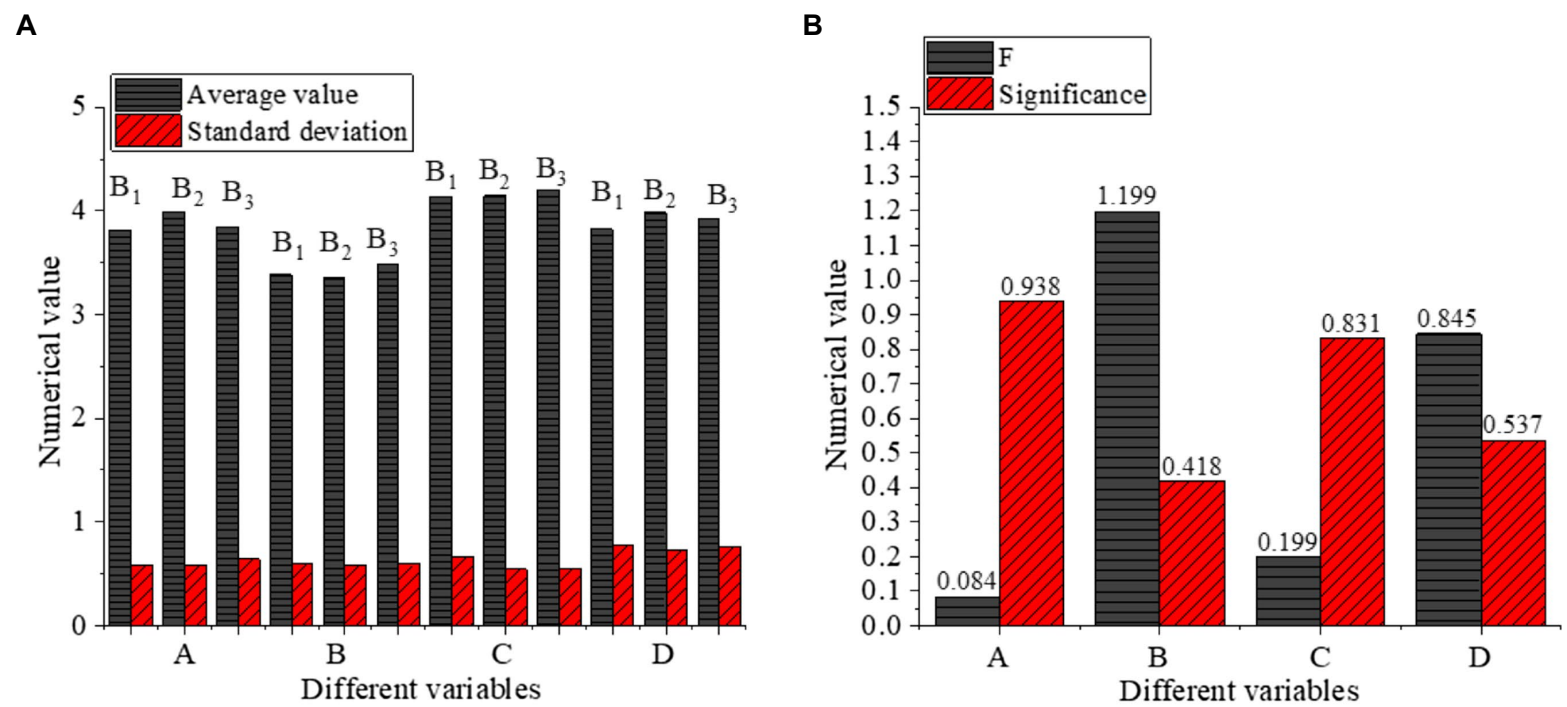
One-way ANOVA of educational background (**A**: different variables with Numerical value, **B**: different variables with Numerical value, B1: Junior college or below B2: Bachelor, B3: Master, A: Cross-cultural competency, B: Cross-cultural adaptation, C: Entrepreneurial intention, D: Psychological adaptation of venture entrepreneurs).

The influence of educational background on different variables is shown in Figure 8.

Figure 8 reveals that the average values of cross-cultural competence, cross-cultural adaptation, entrepreneurial intention, and psychological adaptation of venture entrepreneurs under different education backgrounds of junior college or below, bachelor, and master degree are between [3.3528,4.1986]. The significance of cross-cultural competence, cross-cultural adaptation, entrepreneurial intention, and psychological adaptation of venture entrepreneurs in the single factor of educational background is 0.938, 0.418, 0.831, and 0.537, respectively, Thus, there is no significant difference in the four variables among venture entrepreneurs with different educational backgrounds.

**Figure 9:**
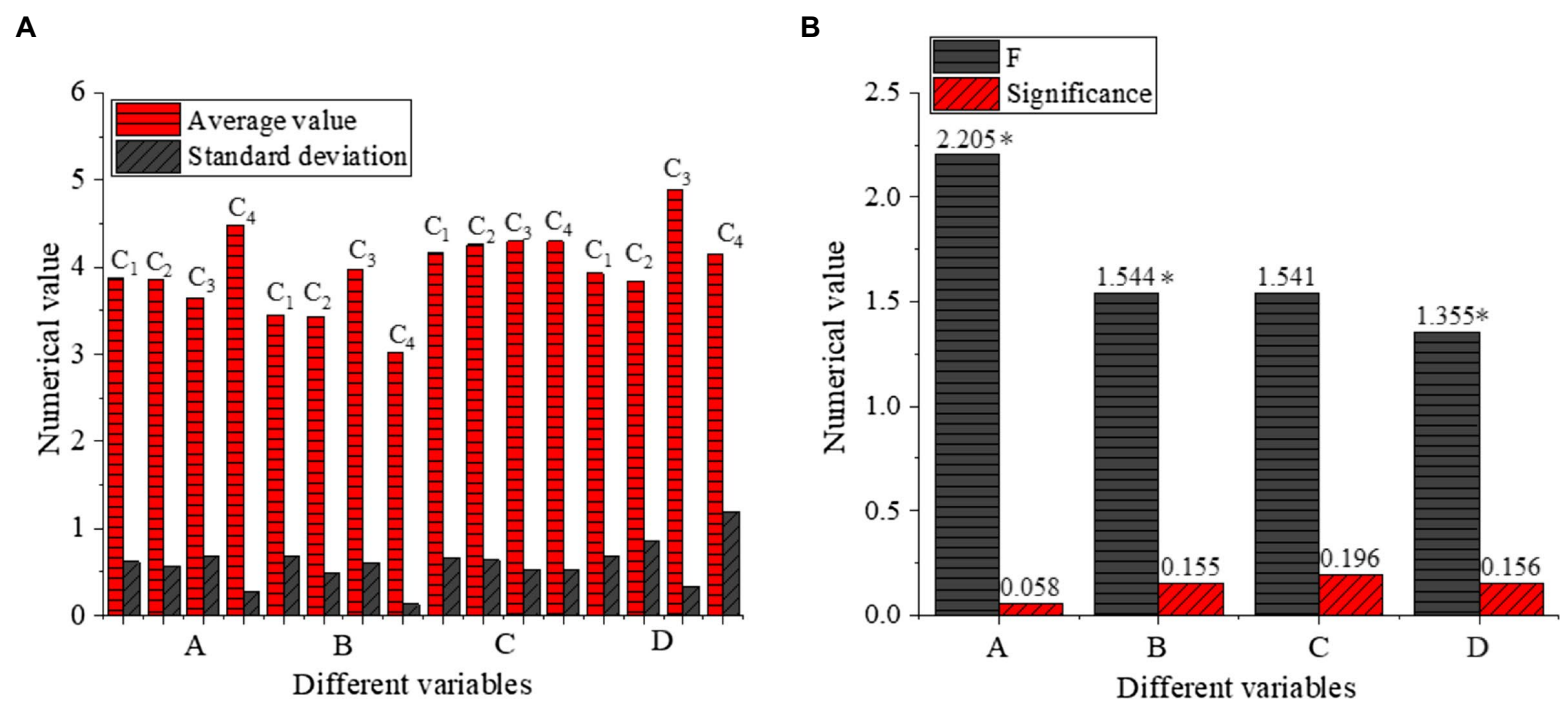
One-way ANOVA of the length of service (**A**: different variables with Numerical value, **B**: different variables with Numerical value, C1: Below one-year, C2: 1–5 years, C3: 5–10 years, C4: 10–20 years, A: Cross-cultural competence, B: Cross-cultural adaptation, C: Entrepreneurial intention, D: Psychological adaptation of venture entrepreneurs *p<0.05).

The influence of the length of service on different variables is shown in Figure 9.

Figure 9 illustrates that the average value of cross-cultural competence, cross-cultural adaptation, entrepreneurial intention, and psychological adaptation of venture entrepreneurs with different of the length of services is between [3.0177,4.8887]. There is no significant difference in psychological adaptation among venture entrepreneurs with different lengths of service, but there is a significant difference in cross-cultural competence, cross-cultural adaptation, and entrepreneurial intention (*p*<0.05).

**Figure 10:**
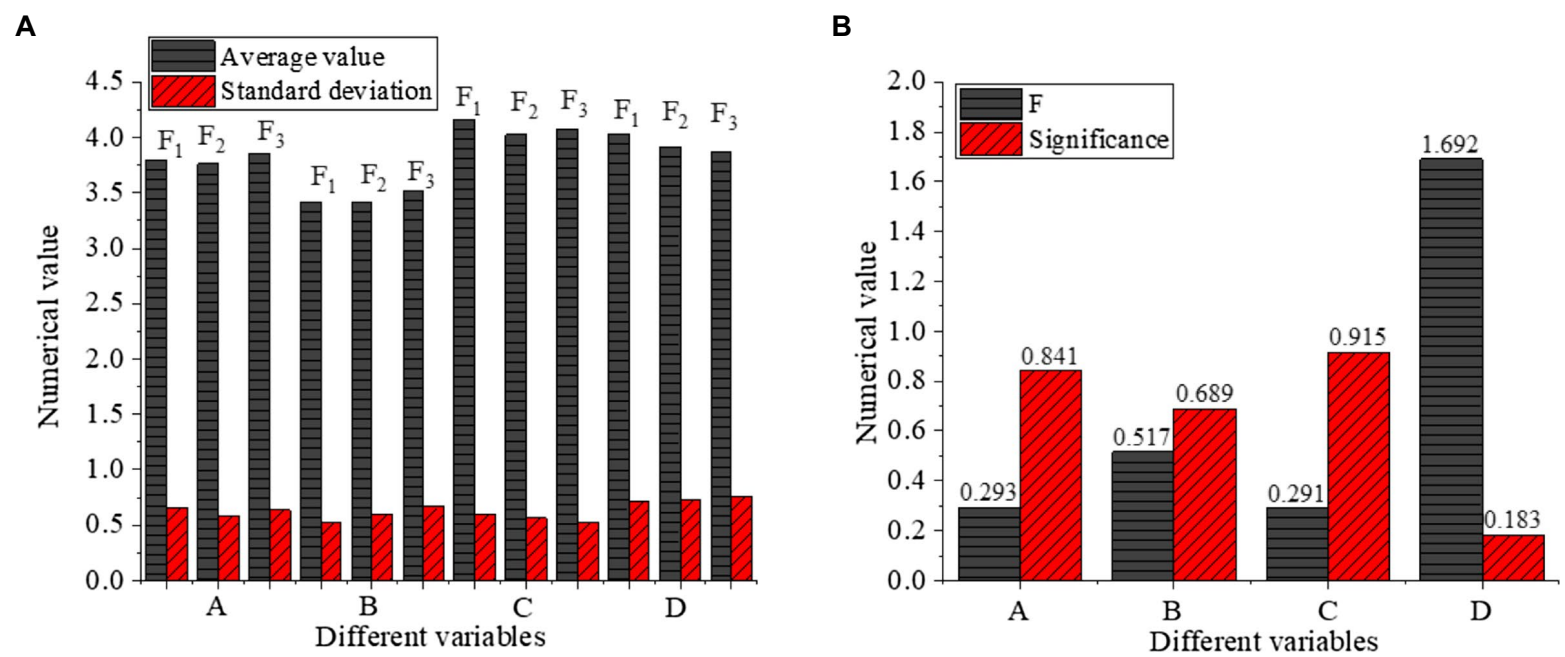
The results of one-way ANOVA of enterprise nature (**A**: different variables with Numerical value, **B**: different variables with Numerical value, F1: Sino-foreign joint venture, F2: Foreign enterprise, F3: Chinese enterprise, A: Cross-cultural competence, B: Cross-cultural adaptation, C: Entrepreneurial intention, D: Psychological adaptation of venture entrepreneurs).

The influence of enterprise nature on different variables is shown in Figure 10.

Figure 10 illustrates that the significance of enterprise nature on cross-cultural competence, cross-cultural adaptation, entrepreneurial intention, and psychological adaptation of venture entrepreneurs is 0.841, 0.689, 0.951, and 0.183, respectively, indicating that enterprise nature has no significant influence on the four variables.

In the process of cross-cultural adaptation, venture entrepreneurs will have social and cultural pressure, work pressure, and psychological adaptation problems, which will have a greater impact on the entrepreneurial intention of entrepreneurs, so the four hypotheses are verified. Social pressure and loneliness are the most significant psychological adaptation problems of venture entrepreneurs during entrepreneurship, mainly due to the lack of deep-seated interpersonal communication and a sense of belonging. Meanwhile, Jena (2020) believed that entrepreneurs’ psychological adaptation problems were mainly caused by the lack of belongingness, which proves that sense of belonging significantly influences psychological adaptation. Differently, the research results here also indicate that the sense of belonging in entrepreneurs’ psychological adaptation is reflected in life and in work. Therefore, venture entrepreneurs should pay attention to the psychological adaptation in the process of entrepreneurship, thereby improving the entrepreneurial performance in the process of cross-cultural adaptation.

## Conclusion

Here, the theoretical basis of cross-cultural adaptation is analyzed in detail based on cultural psychology, and then, the factors of cross-cultural competence, cross-cultural adaptation, entrepreneurial intention, and psychological adaptation of venture entrepreneurs in cross-cultural adaptation are studied through a QS design. The results show that there is a significant relationship between cross-cultural competence, cross-cultural adaptation, entrepreneurial intention, and psychological adaptation of venture entrepreneurs, While the three variables (cross-cultural competence, cross-cultural adaptation, and entrepreneurial intention) are only significantly different in the length of service. Specifically, for venture entrepreneurs, the longer their length of service is, and the older their ages are, the stronger their cross-cultural adaptation ability is, and the smaller the negative impact of cross-cultural adaptability is on their entrepreneurial psychology and entrepreneurial intention. There are also some shortcomings. The psychological adaptation analysis has not fully considered all the dimensions, such as life stress, work stress, social stress, and loneliness. In the latter study, the dimensions of psychological adaptation will be refined, thus making the results more comprehensive.

## Data Availability Statement

The raw data supporting the conclusions of this article will be made available by the authors, without undue reservation.

## Ethics Statement

The studies involving human participants were reviewed and approved by Shaoguan University Ethics Committee. The patients/participants provided their written informed consent to participate in this study.

## Author Contributions

All authors listed have made a substantial, direct and intellectual contribution to the work, and approved it for publication.

## Conflict of Interest

The authors declare that the research was conducted in the absence of any commercial or financial relationships that could be construed as a potential conflict of interest.

## Publisher’s Note

All claims expressed in this article are solely those of the authors and do not necessarily represent those of their affiliated organizations, or those of the publisher, the editors and the reviewers. Any product that may be evaluated in this article, or claim that may be made by its manufacturer, is not guaranteed or endorsed by the publisher.
